# *nfi-1 *affects behavior and life-span in *C. elegans *but is not essential for DNA replication or survival

**DOI:** 10.1186/1471-213X-5-24

**Published:** 2005-10-20

**Authors:** Elena Lazakovitch, John M Kalb, Reiko Matsumoto, Keiko Hirono, Yuji Kohara, Richard M Gronostajski

**Affiliations:** 1 Dept. of Biochemistry, SUNY at Buffalo, 140 Farber Hall, 3435 Main St., Buffalo, NY, 14214, USA; 2 Dept. of Biology, Canisius College, Buffalo, NY, USA; 3 CREST and Gene Network Lab, National Institute of Genetics, Mishima, Japan

## Abstract

**Background:**

The Nuclear Factor I (one) (NFI) family of transcription/replication factors plays essential roles in mammalian gene expression and development and in adenovirus DNA replication. Because of its role in viral DNA replication NFI has long been suspected to function in host DNA synthesis. Determining the requirement for NFI proteins in mammalian DNA replication is complicated by the presence of 4 NFI genes in mice and humans. Loss of individual NFI genes in mice cause defects in brain, lung and tooth development, but the presence of 4 homologous NFI genes raises the issue of redundant roles for NFI genes in DNA replication. No NFI genes are present in bacteria, fungi or plants. However single NFI genes are present in several simple animals including *Drosophila *and *C. elegans*, making it possible to test for a requirement for NFI in multicellular eukaryotic DNA replication and development. Here we assess the functions of the single *nfi-1 *gene in *C. elegans*.

**Results:**

*C. elegans *NFI protein (CeNFI) binds specifically to the same NFI-binding site recognized by vertebrate NFIs. *nfi-1 *encodes alternatively-spliced, maternally-inherited transcripts that are expressed at the single cell stage, during embryogenesis, and in adult muscles, neurons and gut cells. Worms lacking *nfi-1 *survive but have defects in movement, pharyngeal pumping and egg-laying and have a reduced life-span. Expression of the muscle gene Ce titin is decreased in *nfi-1 *mutant worms.

**Conclusion:**

NFI gene function is not needed for survival in *C. elegans *and thus NFI is likely not essential for DNA replication in multi-cellular eukaryotes. The multiple defects in motility, egg-laying, pharyngeal pumping, and reduced lifespan indicate that NFI is important for these processes. Reduction in Ce titin expression could affect muscle function in multiple tissues. The phenotype of *nfi-1 *null worms indicates that NFI functions in multiple developmental and behavioral systems in *C. elegans*, likely regulating genes that function in motility, egg-laying, pharyngeal pumping and lifespan maintenance.

## Background

We are studying the role of the highly conserved Nuclear Factor I (NFI) family of site-specific DNA-binding proteins in metazoan development. NFI was first identified as a protein from human HeLa cells required for efficient adenovirus (Ad) DNA synthesis *in vitro *[1]. A binding site for NFI proteins in the Ad origin of replication is essential for viral replication both *in vitro *and *in vivo *[1-5]. These and other studies suggested that NFI proteins may function in the replication of host cell DNA [6,7]. However there is no direct evidence to support or refute a role for NFI proteins in host DNA synthesis. In contrast, many studies have identified NFI binding sites in the promoter and distal control regions of cellular genes, and deletion analysis of sites shows that NFI proteins are important for gene expression in a variety of cell types [8,9].

Four conserved NFI genes are present in vertebrates (*NFIA*, *NFIB*, *NFIC *and *NFIX *in humans; *Nfia*, *Nfib*, *Nfic *and *Nfix *in mice) [10-13]. Single NFI genes have been identified in the simple metazoans *Amphioxus*, *C. elegans*, and *Drosophila *[14,15], but no NFI genes are present in fungi, *Arabidopsis *or any sequenced prokaryotic genome. Thus the NFI gene family arose during early metazoan evolution and appears to be present only in multicellular animals. NFI proteins bind as either homo- or heterodimers [16,17] to the symmetric consensus sequence TTGGC(N_5_)GCCAA in duplex DNA [18,19]. NFI proteins also bind with lower affinity to sites containing a single TTGGC motif [20,21]. NFI homo- and heterodimers bind to the same sites with similar affinities, making it difficult to determine which family members play essential roles at specific cellular promoters. In addition, the 4 NFI genes in vertebrates are alternatively spliced [16,22] and are expressed in specific but widely overlapping patterns during embryogenesis and adult life [13] making it difficult to assess the role of specific NFI genes in development. The role of the NFI genes in development is of particular interest because binding sites for NFI proteins have been identified in genes expressed in virtually every tissue and organ system of vertebrates including brain [23,24], muscle [25] and other tissues. NFI proteins have also been implicated in the control of gene expression by a number of hormones and physiological modulators including glucocorticoids [26,27], insulin [28,29], TGFβ [30,31] and others.

To assess the roles of NFI genes in development we began a genetic analysis of the NFI genes in mice and *C. elegans*. Disruption of *Nfia *results in neurological defects including agenesis of the corpus callosum, loss of midline glial cells [32], hydrocephalus and perinatal lethality [33]. Disruption of *Nfic *causes defects in tooth development [34] while loss of *Nfib *results in perinatal lethality due to defects in lung maturation [35,36]. In each knockout defects are seen in the presence of the other three vertebrate NFI genes, suggesting that the 4 mouse NFI genes each have essential roles in development. However the presence of 4 NFI genes in mice has made it impossible to test whether NFI activity per se is essential for survival.

Since *C. elegans *has only one NFI gene (*nfi-1*), it provides an ideal system to assess the role of NFI in DNA replication and simple animal development. We show here that *C. elegans nfi-1 *and its products share many properties with their vertebrate homologs including similar DNA-binding activity, alternative splicing and expression during embryogenesis and in adult tissues. Loss of *nfi-1 *results in viable worms with multiple defects in locomotion, pharyngeal pumping and egg-laying, and a shortened life-span. Thus, *nfi-1 *is non-essential for survival but plays an important role in *C. elegans *physiology.

## Results

### Alternative splicing and promoter of the *nfi-1 *gene

**Figure 1:**
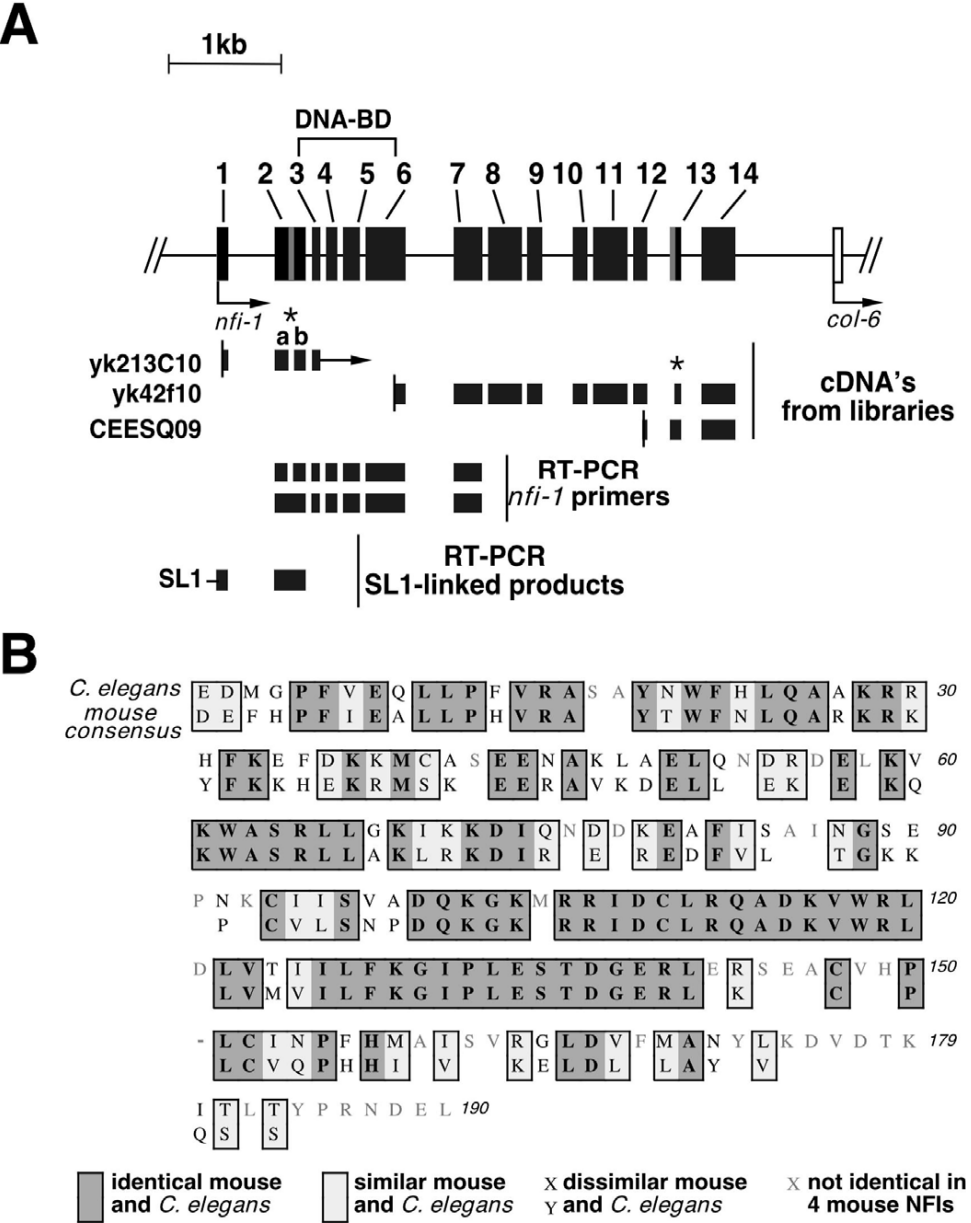
**A) Alternatively spliced products of the *nfi-1 *gene**. The *nfi-1 *gene is shown as a line with exons as numbered solid boxes and alternatively spliced exons as gray boxes. Arrows show the direction of transcription. Below the line are cDNAs from the Kohara and TIGR libraries; the vertical bar indicates the 5' end of the cDNA. In yK213C10 the letters a and b above exon 2 denote that it is alternatively spliced generating 2a and 2b and the arrow on the right denote undetermined sequence in the cDNA. The asterisk (*) on yk42f10 denotes an alternative 3'splice acceptor site in exon 13 used in yk42f10 but not in CEESQ09. Below are depictions of cDNAs obtained by RT-PCR from total and polyA+ RNA of whole worms or isolated eggs using *nfi-1 *exon-specific primers (RT-PCR *nfi-1 *primers). Lastly we show cDNA obtained by PCR using SL1 primer and *nfi-1 *exon-specific primers (RT-PCR SL1-linked products). Some of the alternatively spliced cDNAs have been described previously [14]. **B) Comparison of CeNFI-DBD with the consensus mouse NFI DBD.**The sequence of the CeNFI-DBD (top line) is aligned to a consensus sequence from the 4 mouse NFI-DBDs (bottom line). Gaps in the mouse consensus indicate residues that are not identical between the 4 mouse NFI-DBDs. The dash in the 6^th ^aligned row indicates a single insertion in the CeNFI-DBD sequence needed to align it with the mouse consensus. Dark gray boxes show identical residues in CeNFI-DBD and mouse NFI-DBDs, light gray boxes show residues not identical but similar between CeNFI and mouse NFI-DBDs, unboxed residues with black letters show residues that are not similar between the CeNFI and mouse DBDs, and gray letters in the CeNFI-DBD above gaps in the mouse consensus indicate positions where the 4 mouse genes are not identical. 151 of 190 residues are identical in all 4 mouse NFI-DBDs while 60 of these 151 residues are different in the CeNFI-DBD and 27 of these 60 differences are non-conservative substitutions. The alignment was done in Macvector 6.5.3 using the ClustalW similarity matrix.

The *C. elegans *nuclear factor I gene (*nfi-1*) was first identified by the *C. elegans *sequencing consortium by its homology to mammalian NFI genes [14]. Only a single NFI gene is present in the *C. elegans *genome (Fig. 1A) while vertebrates possess 4 NFI genes. To confirm the structure of the *nfi-1 *gene, we obtained cDNAs from the Kohara and TIGR libraries and made primers within predicted exons for production of cDNAs from RNA of adult worms and purified eggs. These cDNAs confirmed the presence of 14 exons and showed alternative splicing of exons 2 and 13 (Fig. 1A). The differential splicing pattern of *nfi-1 *indicates that different isoforms may be expressed in different cell types. Primary transcripts from most *C. elegans *genes are trans-spliced to SL1 leader sequences and detection of SL1-linked transcripts is frequently used to assess the initially transcribed exons of genes [37]. We confirmed the start site of the gene using PCR with an SL1 primer for SL1-containing transcripts and a primer in exon 2.

### DNA binding by CeNFI is indistinguishable from that of human NFI-C

**Figure 2:**
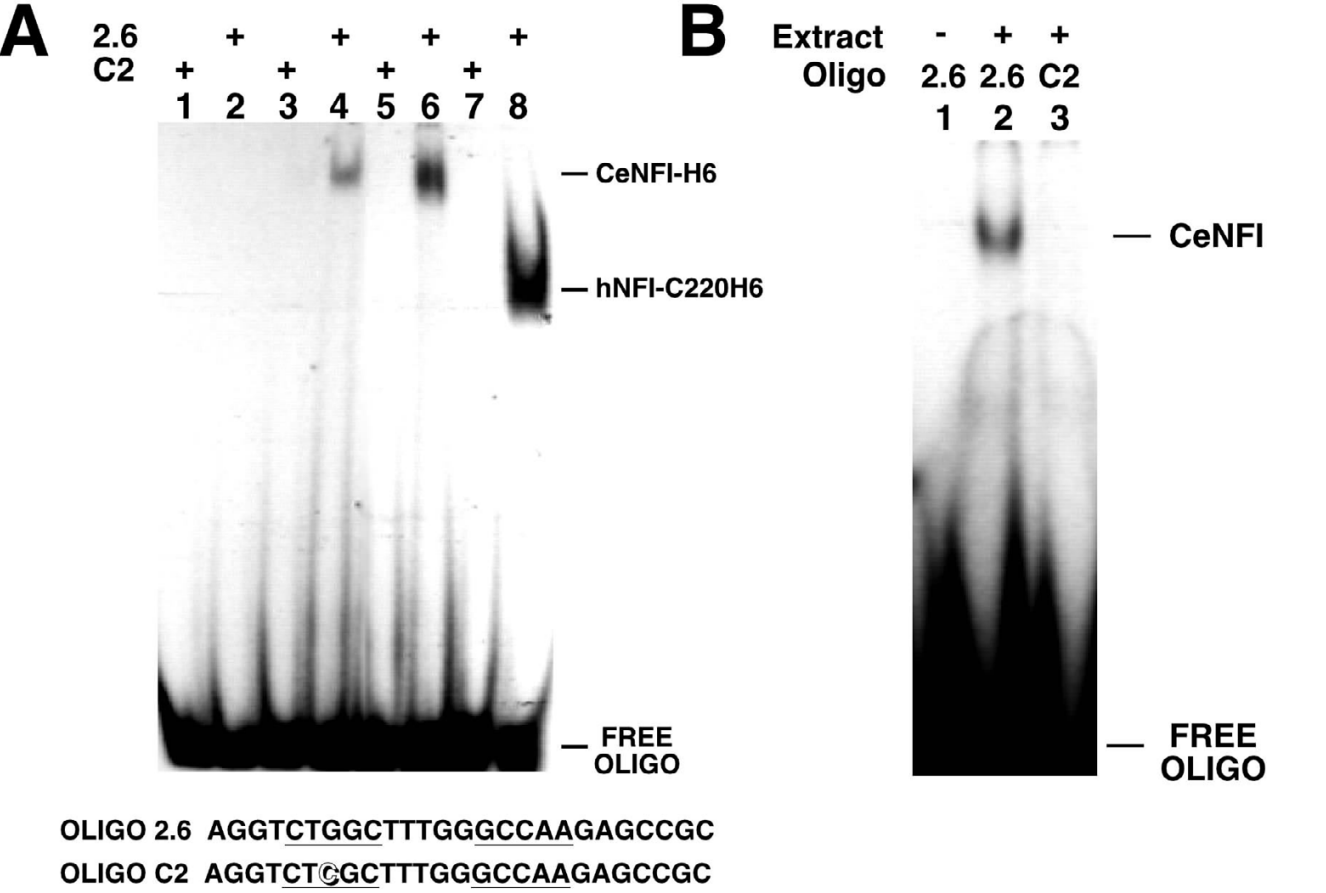
**A) Specific NFI DNA-binding activity of recombinant CeNFI-H6**. Partially purified recombinant H6-tagged CeNFI (containing parts of exons 2–8) and human NFI-C220 were incubated with a duplex oligonucleotide containing an NFI binding site (2.6, even lanes) or an oligo with a single point mutation that abolishes NFI binding (C2, odd lanes) and analyzed on a 6.5% non-denaturing polyacrylamide gel. Lanes 1 and 2, crude *E. coli *extract (neg. control); lanes 3 and 4, ~5 ng partially purified CeNFI-H6; lanes 5 and 6, ~40 ng partially purified CeNFI-H6; lanes 7 and 8, ~5 ng purified human NFI-C220H6. See bottom of panel for sequences of oligonucleotides. **B) Specific NFI DNA-binding activity in worm extracts**. Nuclear extracts of a mixed population of *C. elegans *were prepared and used in a gel mobility shift assay with an oligonucleotide that contains an NFI-binding site (lanes 1 & 2, 2.6) or the same oligo with a single point mutation that abolishes NFI binding (lane 3, C2). Lane 1, no extract; Lanes 2 and 3, *C. elegans *extract (~10 μg). See A for sequences of oligonucleotides.

The predicted DNA-binding domain of *nfi-1 *(CeNFI-DBD) shares homology with the vertebrate NFI proteins, however 60 of 151 residues that are completely conserved among the 4 mouse NFI proteins are changed in CeNFI (Fig. 1B). To assess the DNA-binding activity and specificity of CeNFI, the DNA-binding domain (DBD) of CeNFI was cloned in frame with a 6 histidine-tag, expressed in *E. coli*, partially purified by nickel-affinity chromatography, and was used for *in vitro *DNA binding assays. The DNA-binding activity of CeNFI-H6 was indistinguishable from that of the human hNFI-C220H6, with both proteins binding the NFI-site oligo 2.6 (Fig. 2A) but not the C2 oligo containing a point mutation that prevents vertebrate NFI binding [19,38]. To ask if wild-type *C.elegans *contains a protein with similar DNA binding properties as CeNFI-H6, extracts were prepared from a mixed population of worms and tested for binding to the 2.6 and C2 sites. As expected, proteins were detected that bound to the 2.6 but not the C2 site, confirming the binding specificity of CeNFI (Fig. 2B). The specificity of the native and recombinant CeNFI proteins was also measured by competition of binding of the 2.6 oligo with various unlabeled oligonucleotides and was indistinguishable from the specificity of hNFI-C220-H6 (data not shown). Thus despite the differences between CeNFI and vertebrate NFIs in conserved residues of their DNA-binding domains, their DNA-binding activities are indistinguishable.

### Expression of *nfi-1 *in embryo and adults

**Figure 3:**
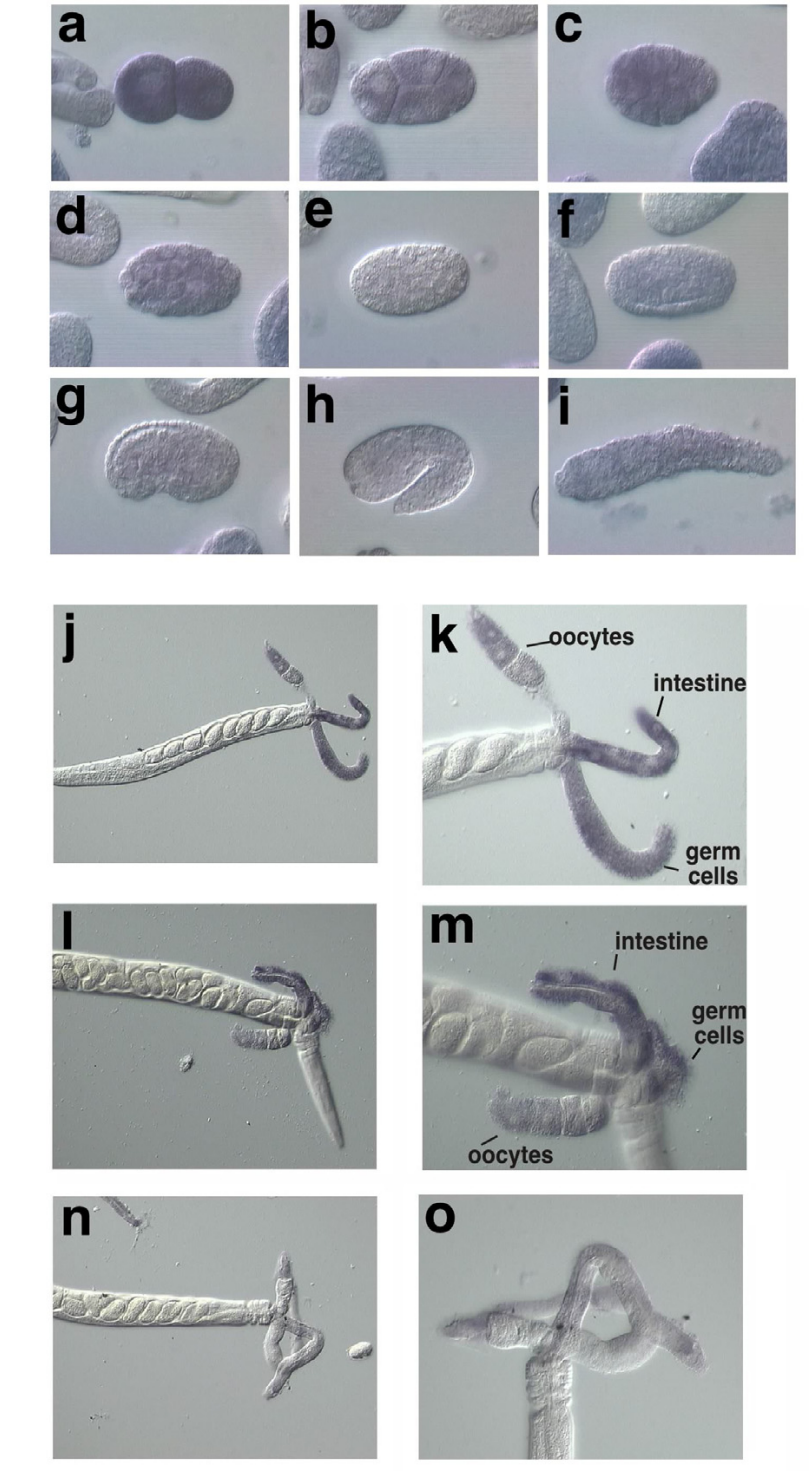
**Expression of endogenous *nfi-1 *mRNA in embryo and adults**. N2 worms were fixed and hybridized with digoxigenin (DIG)-labeled antisense *nfi-1 *probe (from plasmid yk42f10) and bound probe was detected using alkaline phosphatase-conjugated anti-DIG antibodies. Panels: a, 2 cell embryo; b, 4 cell embryo; c, 24 cell embryo; d, beginning gastrulation; e, mid-gastrulation; f, late gastrulation; g, comma stage; h, 1.5-fold stage and i, 2-fold stage. Embryos in a-d and f-i are positive for staining. We are currently investigating the apparent loss of signal at mid-gastrulation (panel e). Panels j-m, antisense probe, adults with exposed internal organs: mature oocytes; intestine; gonadal germ cells; n-o, control sense probe, no specific staining is seen. Right panels k, m, o are two-fold magnifications of those on the left.

In the mouse, the 4 NFI genes are expressed in a complex overlapping pattern during embryogenesis and in adult tissues [13]. To assess the expression pattern of *nfi-1 *transcripts in *C. elegans*, a digoxin-labeled antisense probe from the 3' end of the *nfi-1 *transcript was used for *in-situ *hybridization to fixed embryos and whole worms [39]. *nfi-1 *transcripts are present in the one-cell (not shown) and two-cell stages (Fig. 3a) prior to the onset of zygotic transcription [40,41], indicating that the transcript is maternally inherited. Expression continues in most cells of the embryo throughout early and mid-embryogenesis (Fig. 3b–d) but decreases after gastrulation and no expression is seen in L1 larvae (Fig. 3f–i and data not shown). As expected for a maternally inherited transcript, expression of *nfi-1 *reappears in the adult gonad (Fig. 3j–m). Expression of *nfi-1 *transcripts are also seen in the cytoplasm of gut cells (Fig. 3j–m). No signal is seen using control sense probes (Fig. 3n–o and data not shown). This *in situ *expression pattern indicates that *nfi-1 *could function both early in embryogenesis and in adult worms.

### Isolation of a null allele of *nfi-1*

**Figure 4:**
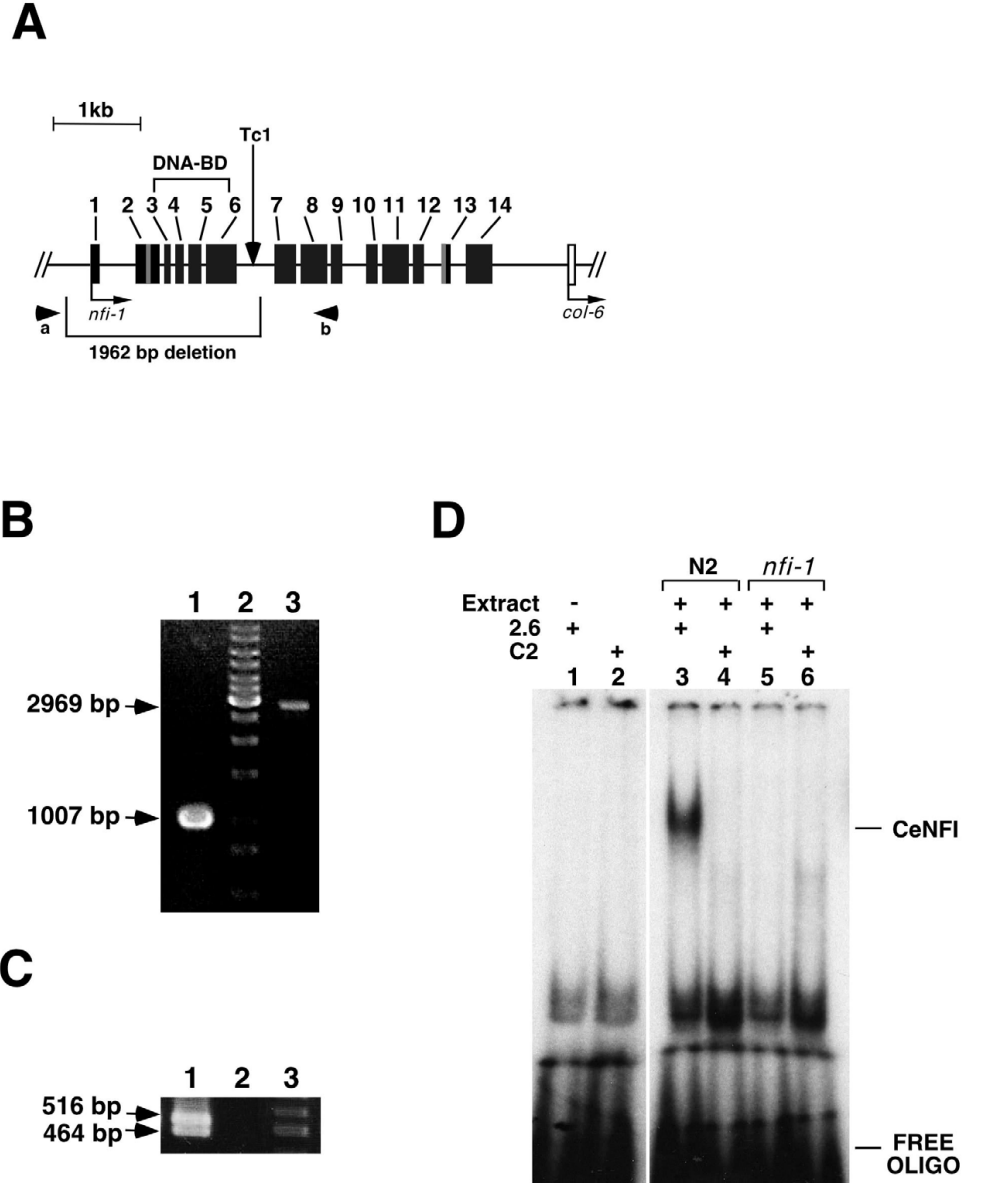
**A) Deletion in C. elegans *nfi-1 *gene**. The relative locations of confirmed exons (boxes) are shown. Square brackets indicate the location of the NFI-1 DNA-binding domain and the region deleted in *nfi-1 *mutant. The arrows indicate the position of the Tc1 insertion in an intron of the *nfi-1 *gene in strain NL747 *pk240 *and locations of PCR primers used in the screening for the deletion. **B) Single-worm PCR reactions on N2 worm and *nfi-1 *homozygous mutant isolated by sib-selection. **The arrows indicate a 1007 bp and 2969 bp PCR products corresponding to the *nfi-1 *mutant (*qa524*) and wild type alleles respectively. Lane 2 is 1 kb DNA ladder. **C) RT-PCR on N2 worms and *nfi-1 *homozygous mutants. **The arrows indicate a 480 bp and 320 bp RT-PCR products amplified using total RNA obtained from N2 worms (lane 1). *nfi-1 *mutants show loss of *nfi-1 *transcripts (lane 2). Lane (3) is 100 bp DNA ladder. **D) Loss of NFI DNA-binding activity in extracts of *nfi-1 *mutants. **Nuclear extracts of a mixed population of N2 worms and *nfi-1 *mutants were prepared and used in a gel mobility shift assay with an oligonucleotide (2.6) that contains an NFI-binding site (lanes 3, 5) or the same oligo with a single point mutation that abolishes NFI binding (C2) (lane 4, 6). See Fig. 2A for sequences of oligonucleotides. Extract of *nfi-1 *mutants show loss of NFI DNA-binding activity (lanes 5, 6). Lanes 1 & 2, no extract.

To assess the role of *nfi-1 *in worms, we isolated a *nfi-1 *mutant using a reverse genetic approach based on the insertion-excision of the transposon Tc1 [42]. Worms carrying an ~2 kb deletion in *nfi-1 *were isolated by PCR screening and sib-selection (Fig. 4A,B). Sequence analysis of the *nfi-1 *transposon excision allele (designated *nfi-1(qa524)*) showed loss of the genomic region corresponding to nucleotides 14754–16715 of cosmid ZK1290. This eliminates the first 6 exons of *nfi-1 *including sequences encoding the DNA-binding domain. RT-PCR shows the absence of *nfi-1 *transcripts in mutant worms (Fig. 4C). A gel mobility shift assay, using nuclear extracts prepared from mix-stage populations of *C. elegans *wild type and *nfi-1 *mutant worms confirmed the loss of CeNFI DNA-binding activity in the mutant worms (Fig 4D). Thus, this mutation in the *nfi-1 *gene is a null allele. The survival of worms homozygous for the null allele shows that *nfi-1 *is not essential for worm survival. The *nfi-1 *mutant allele was backcrossed 12 times to the wild-type N2 strain to remove unwanted mutations prior to assessment of the phenotype.

### Phenotype of *nfi-1 *mutant worms

#### Locomotion defect

**Figure 5:**
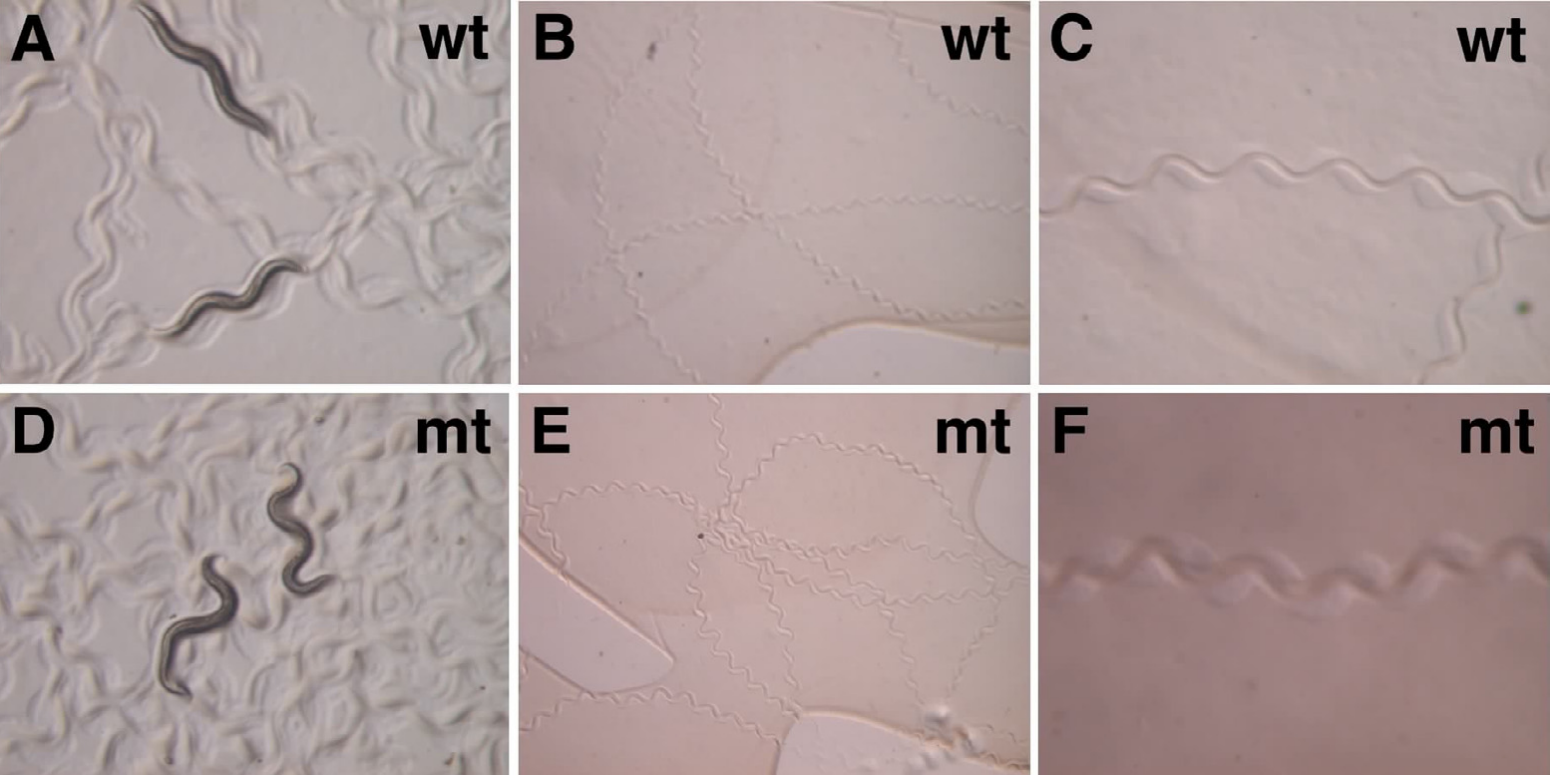
**Locomotion in *nfi-1 *mutants**. Single young adults were spotted in the center of fresh plates and left for 10 min. **(A, D) **Photographs of N2 worms and *nfi-1 *mutants; **(B, E) **Track patterns of N2 worms and *nfi-1 *mutants; **(C, F) **Track patterns of N2 worms and *nfi-1 *mutants with higher magnification. Note less regular tracks in *nfi-1 *mutant vs. N2 worms.

Loss of *nfi-1 *results in a body movement defect (Unc, uncoordinated). *nfi-1 *mutant animals are fairly active and healthy, but are sluggish and flaccid at rest and slightly longer and thinner than N2 worms (Fig. 5A,D). While wild type worms usually move in straight long lines, *nfi-1 *mutant worms often change direction abruptly (Fig. 5B,E). The *nfi-1*mutant worms produce less regular tracks on the bacterial lawn with higher amplitude, and sometimes are slightly coiled when compared to wild-type worms (Fig. 5C,F). This phenotype is more severe in older adults.

#### Egg-laying defect (Egl)

**Figure 6:**
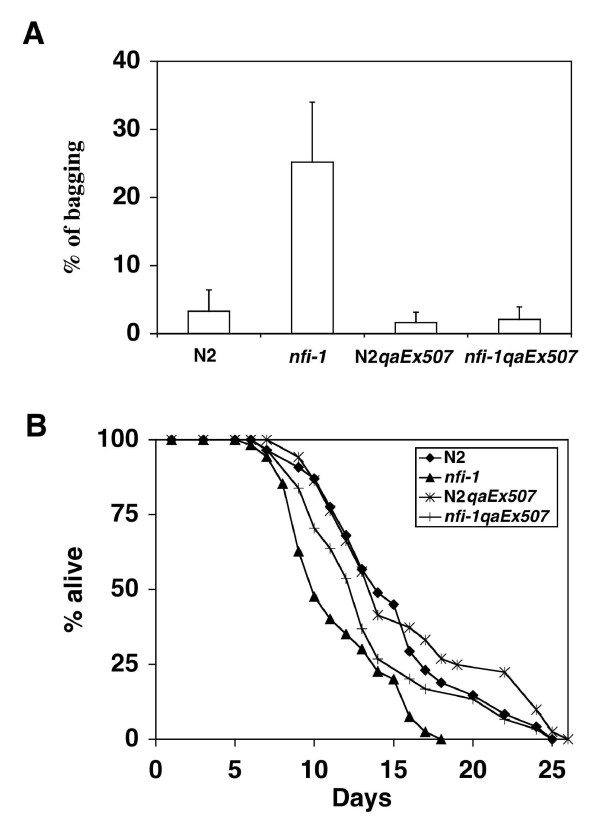
**A) Egg-laying defect in *nfi-1 *mutants and transgenic rescue**. Bagging was measured in wild-type N2, *nfi-1*, N2 worms carrying the transgenic array *qaEx507*(N2 *qaEx507*) and *nfi-1 *worms carrying this array (*nfi-1 qaEx507*). Bars represent % of bagging as the mean of 3–4 independent experiments and error bars show the standard deviation. 30–75 worms of each genotype were scored in each independent experiment. N2 and N2 *qaEx507*showed <5% bagging. The *nfi-1 *mutant worms showed ~30% bagging while the rescued *nfi-1 qaEx507*showed <5% bagging. **B) Shortened life span in *nfi-1 *mutants and transgenic rescue. **Survival curves for the strains described above N2 (n = 57), *nfi-1 *(n = 58), N2 *qaEx507 *(n = 52) and *nfi-1 qaEx507 *(n = 31) are shown. Kaplan-Meier analysis (SPSS11 software) was use to determine median, percentile and p values (log rank test) and Excel was used to construct survival curves. The array generated ~50% rescue of the life-span. The experiment was repeated twice with similar results.

17–35% of older *nfi-1 *mutants have a "bag of worms" phenotype, where the mother is unable to lay fertilized eggs and fills with hatched progeny (Fig. 6A). Appearance and severity of this egg-laying phenotype correlates with the progressive locomotion defect, as young adult *nfi-1 *mutant worms do not bag. In young adults, serotonin stimulated egg-laying in both the wild type and *nfi-1 *mutant animals to a similar extent (data not shown), indicating that the postsynaptic response to serotonin is normal and the contractile apparatus for egg-laying is intact in *nfi-1 *mutant worms.

#### Life-span reduction

The *nfi-1 *mutant worms have a median life span of 10.00 ± 0.69 days as compared to 14.00 ± 0.76 days for wild type worms (p < 0.001) (Fig. 6B). Mutant worms become progressively more sluggish and flaccid as they age. We are currently investigating whether this lifespan reduction is due to the apparent progressive muscle weakness and partial paralysis seen or to a direct effect on known ageing pathways [43-45].

#### Pharyngeal pumping rate defect

Since the Unc and Egl phenotypes of *nfi-1 *mutant worms could reflect aberrant muscle function, we examined another process that can influenced by muscle defects, pharyngeal pumping rate. Pharyngeal pumping rates are reduced in *nfi-1 *mutant worms (Table 1, Days 1–4), with more severe reductions in older vs. younger adult animals (Table 1, Days 3&4 vs. Day 1).

**Table 1:** Rescue of pharyngeal pumping defect by *nfi-1 *transgene

Day^a^	N2	*nfi-1*	N2 *qaEx507*^b^	*nfi-1 qaEx507*^b^
1 (n = 10)	241 ± 5	228 ± 9^c^	243 ± 5	241 ± 8^e^
2 (n = 12)	242 ± 10	187 ± 64^d^	237 ± 8	195 ± 78^d^
3 (n = 12)	214 ± 18	125 ± 79^c^	183 ± 78	200 ± 72
4 (n = 12)	160 ± 62	92.5 ± 86^d^	183 ± 79	133 ± 90

^a ^Pharyngeal pumping was counted for one minute starting on day one, when animals first reached adulthood, for four consecutive days.

^b ^N2 and *nfi-1 *mutant worms were transformed with a transgene containing the *nfi-1 *gene and upstream promoter and *rol-6 *(*qaEx507*).

^c ^This value is statistically significantly different from N2 (P < 0.01)

^d ^This value is statistically significantly different from N2 (P < 0.05)

^e ^This value is statistically significantly different from *nfi-1 *(P < 0.01)

### Rescue of *nfi-1 *mutant with *nfi-1 *transgene

Transgenic rescue was performed to test whether loss of the *nfi-1 *gene was responsible for the observed phenotypes. Transgenic strain XA512 *qaEx507 *was made by injecting a plasmid containing a 10 kb region of genomic DNA including the *nfi-1 *coding region and ~4 kb of upstream promoter region together with a *rol-6(gf) *expressing plasmid into the gonads of N2 worms. We crossed the resulting transgenic array into *nfi-1 *mutant worms to produce strain XA550 *nfi-1(qa524) qaEx507*. Egg-laying was completely rescued in *nfi-1 qaEx507 *worms when compared to the *nfi-1 *mutant worms (Fig. 6A). In addition, the median life span in *nfi-1 qaEx507 *worms of 13.0 ± 0.7 days was significantly longer than the 10.0 ± 0.7 day life span of *nfi-1 *mutant worms (p < 0.05), but slightly less than the 14.0 ± 0.8 day life span of N2 worms (Fig. 6B). The N2 *qaEx507 *stain used as a control has a 14.00 ± 0.49 day median life span, identical to that in non-transgenic N2 worms. The pharyngeal pumping defect was also partially rescued by transgenic expression of *nfi-1 *(Table 1, *nfi-1 qaEx507 *vs. *nfi-1*). The *nfi-1 *transgene had little or no effect on pumping rates in N2 worms (N2 *qaEx507 *vs. N2). These data provide a well-defined developmental system affected by loss of *nfi-1 *that can be examined for cell-autonomous or inductive roles of *nfi-1*. The presence of the *rol-6 *marker gene prevented scoring of rescue of the locomotion phenotype in *nfi-1 qaEx507 *worms.

**Figure 7:**
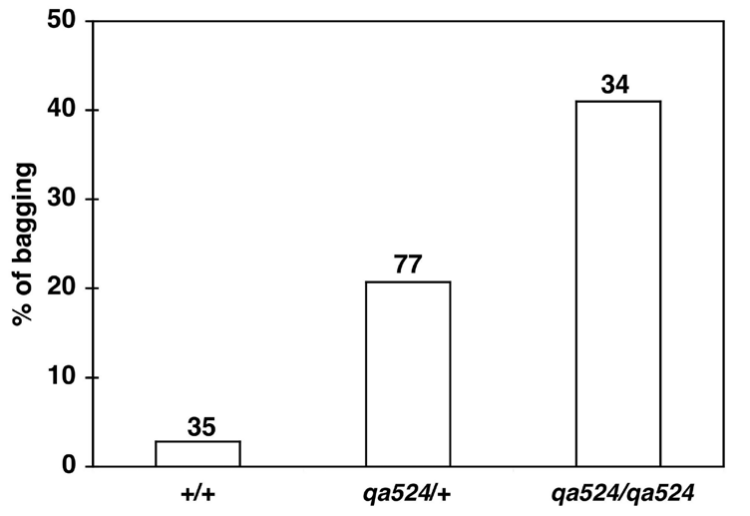
**Haploinsufficiency of *nfi-1 *locus**. Egg-laying defect in progeny of *nfi-1 *heterozygous mutant animals are shown. Bagging was scored in all progeny of two *nfi-1*(*qa524*/+) heterozygous worms (n = 146) derived from eggs laid over 7 hours. All worms were genotyped by single-worm PCR. Bars represent % of bagging in wild type (+/+), *nfi-1 *heterozygous (*qa524*/+) and *nfi-1 *homozygous worms (*qa524/qa524*). The number of worms scored of each genotype are shown above the bars.

Our *in situ *hybridization data indicate that *nfi-1 *transcripts are provided maternally. To test whether maternal *nfi-1 *transcripts could rescue the *nfi-1 *Egl phenotype, *nfi-1 qa524/qa524*, *nfi-1 qa524*/+ and +/+ progeny of heterozygous *nfi-1 qa524/+ *parents were scored for the egg-laying defect (Fig. 7). Bagging was seen in 41% of the resulting *nfi-1 qa524/ qa524 *worms, in 20.7% of *nfi-1 qa524/*+ worms but in <3% of +/+ animals. These data indicate the absence of maternal transcript rescue and possible haploinsufficiency at the *nfi-1 *locus. Thus, transgenic replacement of *nfi-1 *yields either partial (pumping rate and lifespan) or complete (egg-laying) rescue of the phenotypes seen in the *nfi-1 *mutant worms whereas maternal *nfi-1 *transcripts are insufficient to rescue the egg-laying defect.

### Ce titin expression is reduced in *nfi-1 *mutant worms

We used cDNA microarrays to identify genes whose expression is affected by loss of *nfi-1*. Such genes could be either direct or indirect targets of *nfi-1*. Poly A+ RNA was purified from wild type and *nfi-1 *mutant synchronized gravid adults, labeled, and used to probe DNA microarrays containing ~17,000 *C. elegans *genes (Stanford Microarray Database). We analyzed RNA from gravid adults because the phenotype differences between *nfi-1 *mutant and wild type animals are clearer in adults than at earlier stages. The *nfi-1 *gene was scored as the most down-regulated gene in *nfi-1 *mutant worms in all experiments (data not shown). Several dozen genes showed small apparent reductions or increases in levels (2–3 fold) in mutant adults (data not shown) but only one gene, *C. elegans *titin (Ce titin) showed larger changes.

**Figure 8:**
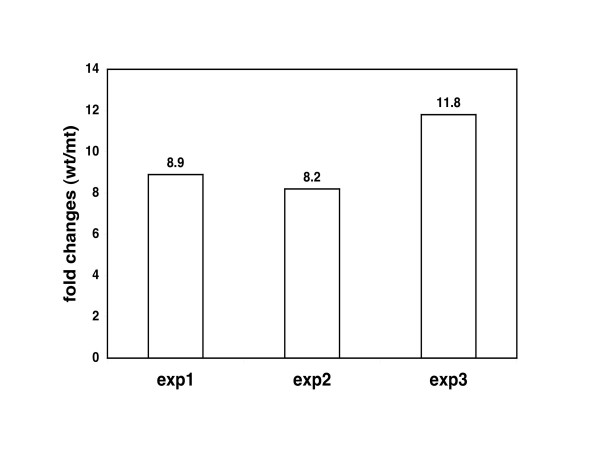
**Down regulation of Ce titin expression in *nfi-1 *mutants assessed by QPCR**. Bars represent fold changes in Ce titin transcript level in wild type N2 vs. *nfi-1 *mutant worms. RNA samples were obtained from 3 independent synchronized adult worm populations for each genotype.

Ce titin (also known as *tag-58*, temporarily assigned gene 58) was predicted to be 5.7-fold lower in mutant worms by microarray analysis. Quantitative PCR confirmed that Ce titin is reduced 8–11 fold in adult *nfi-1*mutant worms (Fig. 8). To date this is the gene that shows the largest decrease in expression in *nfi-1 *mutant worms. A search of the Ce titin gene reveals no overabundance of NFI binding sites (data not shown). Also, a CeTPro transgene expressing a translational fusion of GFP to the 5'-end of the Ce titin gene [46] appears to be expressed at similar levels in WT and *nfi-1 *mutant worms (data not shown). It will be important in future studies to determine whether Ce titin is a direct or indirect target of *nfi-1 *and the possible role of Ce titin in the phenotypes observed.

### Expression of *nfi-1::GFP *reporter transgenes

**Figure 9:**
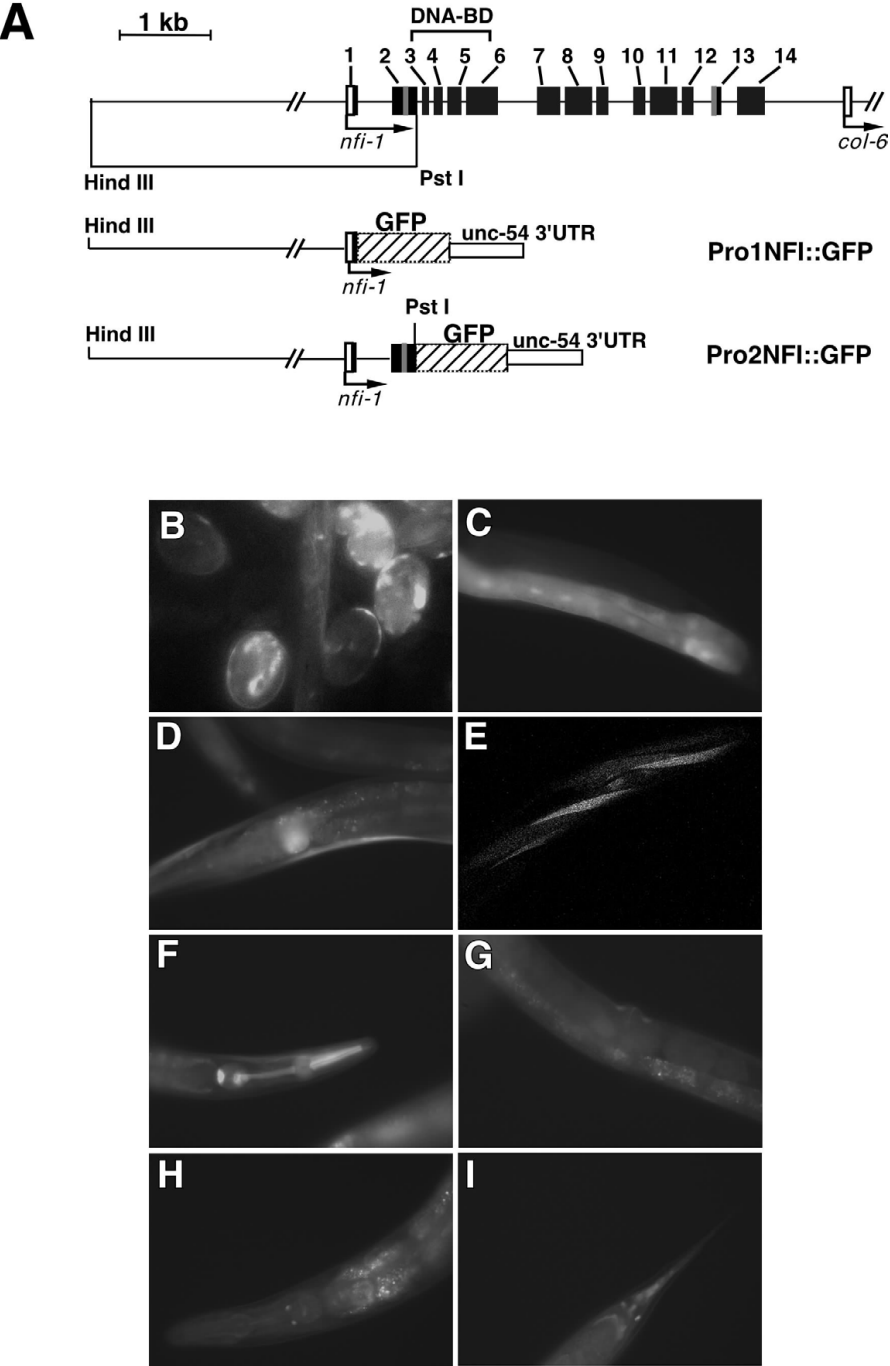
**Expression pattern of the *nfi-1::GFP *reporter transgenes**. The *nfi-1 *locus and structure of the *nfi-1-GFP *fusion constructs are shown **(A)**. *Nfi-1 *coding regions are shown in black, gray boxes indicate alternatively spliced exons, untranslated regions are in white. GFP is shown as a hatched box. Expression of *nfi-1-GFP *reporter constructs was observed in embryos **(B)**, intestinal cells **(C)**, body wall muscles **(D, E)**, pharynx **(F)**, egg-laying muscles **(G)**, several head **(H) **and tail neurons **(I)**. Expression was assessed using a FXA Nikon microscope **(B-D, F-I) **and a Bio-Rad confocal microscope **(E)**.

One limitation of *in situ *hybridization in *C. elegans *is that in older embryos and postembryonic stages it is sometimes not sensitive enough to unambiguously identify individual cells. Since we saw little or no *nfi-1 *expression in muscle by *in situ *hybridization, in an effort to develop a more sensitive assay for *nfi-1 *expression we constructed two GFP-reporter transgenes (Fig. 9A). In *Pro1CeNFI::GFP*, 4 kb of genomic DNA sequence upstream of the *nfi-1 *open reading frame and the sequence encoding the first four residues of the CeNFI protein was fused in frame to GFP. In *Pro2CeNFI::GFP*, the 4 kb promoter region and the sequence encoding the first 94 residues of CeNFI has been fused to GFP. GFP expression for both transgenes was detected in embryos (Fig. 9B). Faint GFP expression is first detected at the late gastrulation stage of embryogenesis (>300 cells) as a diffuse green glow throughout the embryo. By the comma stage expression is detected in many cells along the outer edge of the embryo and expression continues through embryogenesis and is detected in L1-L4 larvae in many of the same cells as in adults. Adult transgenic animals show GFP expression in muscles, neurons and intestinal cells (Fig. 9B–I). Among the muscles, fluorescence was strongest in the pharynx and head muscles, was observed with less frequency in other body wall muscles and was seen occasionally in vulva muscles. Expression was also seen in two pairs of neurons located near the posterior bulb of the pharynx, and in several as yet unidentified tail neurons. Expression patterns in multiple transgenic lines from each reporter were similar with the exception that *Pro2CeNFI::GFP *expression was detected more consistently in head neurons and *Pro1CeNFI::GFP *was seen with higher frequency in body-wall muscles. However expression of both transgenes was mosaic, showing expression in only subsets of cells and animals in each population. Since mosaic expression of GFP was seen in transgenic strains from both arrays, transgenic array *Pro1CeNFI::GFP *was integrated by γ-irradiation. However similar mosaic expression was seen with the integrated array (data not shown). These data may indicate that additional elements are needed for stable regulation of *nfi-1 *expression and that such elements may be located further downstream in the *nfi-1 *genomic sequence.

## Discussion

### *nfi-1 *gene structure and DNA binding properties

These data show that *nfi-1 *is not essential for embryogenesis and thus is not essential for DNA replication. However the multiple defects seen in *nfi-1*-deficient worms, abnormal locomotion, pharyngeal pumping and egg-laying defects, and a reduced lifespan indicate that *nfi-1 *is essential for these developmental and behavioral processes. *nfi-1 *shares a number of properties with the vertebrate NFI genes, including alternative splicing (Fig. 1) and the DNA-binding properties of its protein product CeNFI (Fig. 2B,C). The alternative splicing seen in *nfi-1 *is reminiscent of the complex splicing pattern seen in the vertebrate NFI genes [22]. While the relevance of this alternative splicing in *C. elegans *is unclear, the finding that alternatively spliced forms of vertebrate NFIs have different transcriptional modulation properties [16,47,48] suggests that the multiple *C. elegans *isoforms may also have distinct functions. It will be of particular interest to determine whether alternatively spliced isoforms of *nfi-1 *are differentially expressed in worm cells during development and in adults. In addition, when specific downstream targets of *nfi-1 *are identified it will be important to determine whether the isoforms differ in their ability to modulate gene expression in *C. elegans*.

The sequence conservation of the predicted DNA-binding domain of *nfi-1 *with the vertebrate NFI proteins was the initial indication that *nfi-1 *encodes the single *C. elegans *NFI gene. However among the 4 mouse (and human) NFI genes, 151 of 190 residues of their DNA-binding domains are completely conserved, while 60 of these 151 residues are changed in CeNFI (Fig. 1B). We show that CeNFI binds to the same DNA sequence as hNFI-C and that binding is abolished by a point mutation known to abolish binding by products of the 4 vertebrate NFI genes (Fig. 2) [38]. This raises the question of why there has been so little divergence of the 4 vertebrate NFI DNA-binding domains during evolution. While we have shown similar DNA binding specificities of CeNFI and hNFI-C to a matched set of WT and mutant NFI binding sites, it is possible that subtle differences exist in their DNA-binding specificity or affinity or that the conserved vertebrate residues are more important for binding within the more complex vertebrate genome. Since the 4 vertebrate NFI genes are sometimes expressed in the same cells *in vivo *[13], it is possible that there has been selection for conservation of residues involved in DNA binding due to competition between the 4 NFI gene products, and that this has led to the observed high degree of sequence conservation. Similar high levels of sequence conservation are seen in the DNA-binding domains of all the homologous vertebrate NFI genes from *Xenopus *[49,50] to humans [12,51].

It is possible that the additional conserved residues in the vertebrate NFI DBDs compared to *nfi-1 *confer additional functions to the vertebrate NFI proteins, such as interacting with proteins required for transcriptional modulation specifically in vertebrates, or for the stimulation of Ad DNA replication. Indeed, 2 sets of mutations in the NFI-C DBD that abolish Ad replication without affecting DNA-binding or dimerization are in residues not conserved between NFI-C and CeNFI [52]. However, each of the 4 cysteine residues shown previously to be essential for DNA-binding activity and redox-regulation of DNA-binding activity of vertebrate NFIs are conserved in CeNFI (Fig. 1B, labeled C) [53,54]. The conservation of these cysteine residues is consistent with our observation that the DNA-binding activity of CeNFI is sensitive to reversible inactivation by chemical oxidizing agents such as diamide *in vitro *(data not shown), as is the activity of the vertebrate NFIs [53,54]. Given the divergence of the CeNFI and vertebrate NFI DBDs it will be important to determine whether the *nfi-1 *DBD can substitute for the vertebrate NFI DBDs *in vivo*, and whether the vertebrate NFI DBDs possess equivalent activities in *C. elegans in vivo*.

### Defects in *nfi-1 *mutant worms

Deletion of the *nfi-1 *gene results in a number of behavioral defects, each of which may be related to defects in muscle or neural function. The *C. elegans *hermaphrodite has four major muscles types, body wall, pharyngeal, vulval and enteric [55]. Body-wall muscles and pharyngeal muscles, used in locomotion and pumping food, respectively, function in the processes affected in *nfi-1 *mutants. The structures most often affected in Unc mutants are the body-wall muscles, the nervous system and the hypodermis [56]. Polarized light microscopy showed no obvious deformities in the body wall-muscles of *nfi-1 *mutant animals, such as defects in muscle attachment and overall sarcomere structure. Thus, the muscles appear normal at a gross level. Likewise, we analyzed the expression pattern of the body wall muscle specific *hlh-1*::GFP [57] and muscle specific CeTPro transgenes [46] in the *nfi-1 *deletion mutant background by confocal microscopy and saw no differences between mutant and wild type animals (data not shown). Thus any muscle defects are not due to gross structural changes within the muscle. Many older *nfi-1 *deficient animals display egg-laying defects, suggesting that vulval or/and uterine muscles or their controlling neurons may be affected. The normal stimulation of egg-laying by serotonin suggests that there is no major loss of vulval or uterine muscle function in young adult *nfi-1 *deficient worms [58,59]. We have visually examined the functions of the enteric muscles involved in food movement through the gut and defecation and found no obvious defects in young adults. In older adults, contractions of the enteric muscles were less regular, but it is unclear whether this is an intrinsic defect or related to the defects in pharyngeal pumping seen in older adults.

The absence of obvious muscle defects in *nfi-1 *null worms raises the possibility that neural defects could underlie the phenotypes seen in these animals. For example, *nfi-1 *mutants show a reduced pharyngeal pumping rate, a process regulated primarily by the M3, M4 and MC pharyngeal neurons [60]. Some or all of defects seen in *nfi-1 *mutant worms, mild Unc with flaccidity, pharyngeal pumping and egg-laying are also seen in worms containing single mutations in genes expressed in neurons. For example, some mutations in *egl-30*, a heterotrimeric G_q_α subunit expressed in muscles and neurons, have weak Unc, egg-laying and pharyngeal defects [61]. In addition, some loss of function mutations in the G protein signaling molecule *egl-10 *and the Gβ_5 _ortholog *eat-11 *cause sluggish movement, egg-laying, and pharyngeal pumping defects [62,63]. Since a number of *eat *genes are expressed specifically in neurons, these data indicate that neural dysfunction caused by loss of *nfi-1 *could generate this range of phenotypes. It will be important in future studies to determine whether *nfi-1 *expression is needed neurons, muscles, both cell types or other cell types to alleviate the phenotypes seen in *nfi-1 *mutant worms. While our preliminary QPCR data indicate no changes in the transcript levels of *egl-30*, *egl-10 *or *eat-11 *(N. Butz, unpublished data), it is possible that the activity of the G protein signaling system is affected in *nfi-1 *mutant worms. Thus, it would be useful in future studies to directly test whether *nfi-1 *is involved in G protein signaling in *C. elegans*.

The only gene whose expression has been shown to change in response to loss of *nfi-1 *is the muscle-specific gene Ce titin. This reduction in a muscle-specific gene indicates that muscle defects could contribute to the observed locomotion and other phenotypes. Ce titin is a massive protein, with isoforms in *C. elegans *of 2.2MDa, 1.2MDa and 301KDa [46]. Ce titin is found in the I-bands of larval and adult muscle in worms. Mutations in mouse *titin *genes cause muscular dystrophy [64], cardiac development defects and muscle weakness [65]. Thus, down regulation of Ce titin expression could contribute to the motility and egg-laying defects observed in *nfi-1 *mutants. However, the large size of the Ce titin gene has made it difficult to generate transgenic lines that conditionally express Ce titin. Recent studies have reported that neither existing mutations in Ce titin, nor RNAi experiments, have provided clues as to the function of Ce titin in worms [46]. Thus it will be important in the future to test directly the role of Ce titin in the defects seen in *nfi-1 *mutant worms.

Finally, it is unknown whether the observed motility, egg-laying and pharyngeal pumping defects contribute to the shortened life-span of *nfi-1 *null worms. For example, as pharyngeal pumping becomes more defective the animals could starve, leading to progressive muscle wasting, paralysis and death. However, most severe *eat *mutants have extended lifespans rather than reduced lifespans, most likely due to caloric restriction [66]. These data indicate that the reduced lifespan seen in *nfi-1 *deficient worms could be independent from the pharyngeal pumping defect. In addition, while bagging could reduce apparent lifespan, we eliminated bagged animals from our lifespan analysis so as to exclude bagging as a direct cause of the shortened lifespan. Lastly, the motility defect is seen in young adults a few days prior to bagging and is seen in all adults, even those that do not bag. Thus it appears unlikely that bagging contributes directly to the movement defect. It will be important to use directed expression of *nfi-1 *in different cell types to test the dependence or independence of the observed phenotypes from each other. We are also currently testing whether *nfi-1 *is part of the genetic pathways known to be important in worm aging including the *daf *pathway [67].

### Roles of NFI transcription factors in development

Since NFI genes have been found in all metazoa sequenced to date and multiple NFI genes are present in complex animals [68], it was our initial hypothesis that *nfi-1 *would be essential in worms and that deletion of the gene would be lethal. Lethality would be predicted if *nfi-1 *has an essential role in DNA replication. However, all of the defects observed are late physiological defects and are most severe in older adults. This set of late defects can be compared to the late gestational defects seen with deletion of single NFI genes in mice. Disruption of the mouse *Nfia *gene causes agenesis of the corpus callosum, the loss of specific midline glial populations and perinatal lethality [32,33]. Loss of *Nfic *produces specific defects in tooth development including aberrant incisor formation and failure of root formation in molar teeth [34]. Targeted insertion into the *Nfib *locus causes perinatal lethality due to a failure of late fetal lung maturation [35,36]. One common feature of the defects seen in NFI-deficient mice is that they occur either late in fetal development (*Nfia *and *Nfib*), or early in postnatal development (*Nfic*). While some of the developmental systems affected by the loss of NFI genes in mice are not present in worms (e.g. lungs and teeth), it is possible that the underlying molecular mechanisms disrupted in NFI-deficient mice are also affected by loss of *nfi-1 *in worms. Thus it will be important to test genes identified as important in the phenotypes of NFI-deficient mice for potential roles in the motility, egg-laying and pharyngeal-pumping defects seen in *nfi-1*mutant worms.

## Conclusion

These data show that *nfi-1 *is not essential for worm survival but plays important roles in locomotion, egg-laying, pharyngeal pumping and maintenance of a normal life-span. These are the first data to show that while NFI proteins are essential for adenovirus DNA replication, they are not essential for DNA replication in simple animals.

We show that while many residues of the DNA binding domain of worm NFI differ from those found in vertebrate NFIs, the DNA binding activity and specificity of the worm protein is indistinguishable from that of the vertebrate NFIs. These data suggest that the very strong conservation of many residues in the DNA-binding domains of vertebrate NFIs is not needed for DNA-binding specificity but may serve another function, perhaps for interactions with specific vertebrate proteins. In addition, like the vertebrate NFI genes *nfi-1 *is alternatively spliced, raising the possibility that different CeNFI isoforms may have different biological functions.

The phenotypes of *nfi-1 *null worms are rescued by zygotic expression of *nfi-1 *from its natural promoter but not by maternal transcripts. We propose that *nfi-1 *regulates gene expression directly in cells in which it is expressed and affect the function of these cells. However, the precise cell types in which loss of *nfi-1 *causes these defects in motility, egg-laying, pharyngeal pumping and lifespan is unknown. Directed expression of *nfi-1 *in different cells of the worm should allow us to determine in which cells *nfi-1 *is needed for normal lifespan, motility, egg-laying and pharyngeal pumping.

Future studies will assess whether *C. elegans *can be used as an experimental system to assess the potentially distinct physiological functions of the vertebrate NFI gene products. For example, if the 4 vertebrate NFI genes have segregated the functions of the single worm *nfi-1 *gene into 4 distinct entities, then each vertebrate NFI gene might have distinct properties when expressed in *nfi-1*mutant worms. It will also be important to determine the specific *nfi-1 *target genes in *C. elegans *whose altered expression is responsible for the phenotypes observed, the specific cell types in which *nfi-1 *must be expressed to prevent each phenotype, and to use *C. elegans *genetics to identify genes essential for NFI-regulated transcription.

## Methods

### Strains and transgene plasmids

The Bristol strain N2 was used as wild type and worms were grown at 20°C using standard techniques [69]. Strains containing *hlh-1*:*GFP*, *CeTPro *and NL747 *pk240 *were kindly provided by Michael Krause (NIH/NIDDK/LMB, Bethesda, MD), Guy M. Benian (Emory University School of Medicine, Atlanta, GA) and Ronald Plasterk (Hubrecht Laboratory, Utrecht, The Netherlands), respectively.

For rescue experiments the *nfi-1 *locus from cosmid ZK1290 was cloned into pBluescriptKS+ to generate pCeNFIG. pCeNFIG (25 ng/ul) was injected in N2 wild type worms along with the *rol-6(gf) *marker plasmid pRF4 (125 ng/ul) using standard microinjection procedures to produce the transgenic strain XA512 *qaEx507 *[69]. Transgenic arrays were crossed onto the *nfi-1 *deletion strain (XA549 *nfi-1(qa524)*, see below) screening F1 and F2 progeny by PCR for the wild-type and *nfi-1(qa524) *deletion alleles.

Two GFP-reporter constructs were made to assess *nfi-1 *expression. *Pro1CeNFI::GFP *was made by cloning a PCR-generated HindIII-XmaI fragment of ZK1290 into pPD95.69 (kindly provided by A. Fire) to generate a translational fusion of *nfi-1 *and GFP. The 5'end of the construct was extended by cloning the HindIII-BglII fragment of pCeNFIG into the vector. *Pro2CeNFI::GFP *was made by cloning a HindIII-PstI fragment of pCeNFIG into corresponding sites of pPD95.69. The constructs were coinjected with *rol-6 *marker into N2 worms as described above. Three independent transgenic lines were analyzed for *Pro1CeNFI::GFP *expression and two lines for *Pro2CeNFI::GFP *expression.

A vector expressing the 6his-tagged DNA-binding domain of CeNFI (pCeNFI-H6) in *E. coli *was produced by cloning a fragment of *nfi-1 *cDNA encompassing the predicted DNA-binding domain of *nfi-1 *downstream of a 6 histidine tag into the pET8C vector [70].

### Generation of the *nfi-1 *deletion mutant

*C. elegans *NL747 *pk240 *contains a Tc1 transposon insertion in the 6 th intron of *nfi-1*. The location of the Tc1 element was mapped by genomic PCR and sequencing. 100 populations of worms were established at 15°C on 3 cm NGM/OP-50 plates starting with 10–20 worms each, and the worms were collected in M9 buffer when nearing starvation. One third of the worms were put onto fresh plates and the rest were used to prepare two separate lysates for DNA. Thirteen of the 100 populations showed a strong excision bands by PCR screening (Platinum Taq Polymerase, Invitrogen) with primers 1 (5'GTATTTGTACGACCCTCTGCG) and 2 (5'TGCTGTTGAACGGAATGCACC). To distinguish between germ line and somatic mutations the second lysate for each positive population was screened by PCR. Only two populations showed deletions with both lysates indicating that more than one worm carried the deletion and therefore the deletions were in the germ line. The locations of the deletions were determined by subcloning PCR products into pCRII-TOPO (Invitrogen) and sequencing. A clonal strain of worms carrying one of these deletions was isolated by multiple rounds of PCR screening and sib-selection (strain XA549 *nfi-1(qa524)*). The mutant worms were backcrossed 12 times onto the wild-type N2 strain to remove unwanted mutations. For genotyping, PCR with primers 1 and 3 (5'TCGGAGGAGGTGGTAGACAT) amplified a 623 bp product from the deletion allele and primers 1 and 4 (5'GTGAGTCTTGAGGTGCTTCTG) amplified a 968 bp product from the wild type allele.

### RT-PCR cloning and cDNA isolation

Total RNA was prepared from adult worms or eggs (Trizol, Life Technologies) and was reverse transcribed using oligo dT (Superscript, Life Technologies) according to the manufacturers instructions. Poly A+ mRNA was isolated from total RNA using standard techniques (Oligotex, Qiagen). PCR was performed as described previously [14] using primers from within predicted exons 1–14 of *nfi-1 *or SL1 primers (GGTTTAATTACCCAAGTTTGAG) (*nfi-1 *primer sequences available upon request) and PCR products were cloned into the pCRII-TOPO vector (Invitrogen). DNA from individual clones was isolated (Wizard, Promega) and sequenced (Roswell Park Cancer Institute DNA Sequencing Core) and the sequence obtained compared with that of the predicted exons of *nfi-1 *(*C. elegans *cosmid ZK1290, Genbank Acc. #U21308). The cDNA clones yk42f10 and yk213C10 were from the *C. elegans *cDNA sequencing/expression project (CREST and Gene Network Lab, National Institute of Genetics, Mishima) and CEESQ09 was from The Institute for Genomic Research (TIGR).

### *In situ *hybridization

Endogenous *nfi-1 *transcripts were analyzed as described previously [39], using digoxin-labeled antisense probe for *nfi-1*. Embryos were either freeze-fractured or were treated with chitinase to digest the eggshell prior to hybridization.

### DNA-binding assays

Electrophoretic mobility shift assays for the analysis of NFI protein binding to oligonucleotides was performed as described previously [38,54]. Worm extracts were prepared from dounce homogenized mixed-age worms using the NP40-based extraction buffer described previously [38]. CeNFI-H6 (332aa) and hNFI-C220-H6 (230aa) proteins were produced and partially purified from extracts of *E. coli *as described previously [54]. The labeled oligonucleotides used contained a wild-type NFI binding site (2.6) or a site with a single point mutation (C2) shown previously to abolish the binding of vertebrate NFI proteins [19,38,54,71].

### Behavioral and functional assays

For locomotion assays wild type N2 and *nfi-1 *mutants were raised at 20°C on NGM/OP50 plates. To obtain age-synchronized worms young adults were placed on a bacterial lawn to allow them to lay eggs for 3 hours and then removed. Worms were observed at all larval stages. To photograph worms tracks, single young adult worms were moved to new plates containing a 1 day old bacterial lawn and were left undisturbed for 10 min.

"Bag of worms" phenotype was scored for each strain using age-synchronized worms. Worms were observed each day from the start of egg laying until one day after cessation of egg laying. They were moved to new plates every second day to prevent overcrowding and starvation. Other aspects of egg-laying behavior such as the brood size, stage of newly laid eggs, and response to serotonin were assessed as described previously [58,59]. Brood size was the same in WT and *nfi-1 *null worms.

For life-span assays wild type N2 and *nfi-1 *mutants were raised and synchronized as described above except that 250 μg/ml of fungizone was included to reduce fungal contamination. Worms were maintained at 20°C and adult worms were moved to new plates every second day until progeny production ceased. Worms were observed every day and were scored as dead when they failed to respond to touch. Animals that crawled off the plate or died from internally hatched progeny were censored, but incorporated into the data until the day of disqualification.

Pharyngeal pumping was counted for one minute starting on the first day of adulthood for four consecutive days [60]. Worms were placed on NGM/OP50 plates and left undisturbed for 1 hour before measuring. All animals remained on food during the period of observation.

### Microarray assays and QPCR

Synchronized populations were generated by hatching eggs after alkaline hypochlorite-treatment and the worms were collected as gravid adults. Total RNA was prepared with Trizol (Life Techologies) and poly A RNA was purified using Poly(A)Purist™ kit (Ambion). DNA microarray assays were performed and analyzed in the Stanford Microarray Database (Stanford). cDNA for QPCR was made using random primers and Super-Script (Invitrogen). QPCR was performed using AmpliTaq Gold^R ^with the Gene Amp^R ^SYBR green kit (Applied Biosystems) on a Bio-Rad iCycler. The *inf-1 *gene was used as an internal control for RT-PCR reactions and for normalized quantification in QPCR reactions. Primers used in QPCR are available on request.

## Authors' contributions

EL isolated the *nfi-1 *deletion strain and most transgene plasmids, performed most transgenic generation and phenotype characterization, and helped draft the manuscript. JMK performed microinjections for transgenic rescue and some phenotype characterization and contributed to manuscript preparation. RM performed QPCR. KH performed *in situ *hybridizations and β gal staining. YK identified specific cell types in which *nfi-1 *is expressed. RMG initiated the project and designed some of the experiments, generated some transgenics, produced vectors and proteins used in the study, and finalized preparation of the manuscript.
